# Analytical Model and Numerical Analysis of Composite Wrap System Applied to Steel Pipeline

**DOI:** 10.3390/ma14216393

**Published:** 2021-10-25

**Authors:** Djouadi Djahida, Ghomari Tewfik, Maciej Witek, Mechri Abdelghani

**Affiliations:** 1 Composite Structures and Innovative Materials Laboratory (LSCMI), University of Sciences and Technology USTOMB, BP 1505, Oran 31000, Algeria; djahida.djouadi@univ-usto.dz (D.D.); abdelghani.mechri@univ-usto.dz (M.A.); 2 Aeronautics and Propulsive Systems Laboratory (LASP), University of Sciences and Technology, USTOMB, BP 1505, Oran 31000, Algeria; tewfikghomari@yahoo.com; 3 Gas Engineering Group, Warsaw University of Technology, 20 Nowowiejska St., 00-653 Warsaw, Poland

**Keywords:** composite overwrap repair, defected steel pipe wall, composite sleeve sizing, finite element analysis

## Abstract

Composite overwraps are a cost-effective repair technology, appropriate for corrosion defects, dents, and gouges for both onshore and offshore steel pipelines. The main benefit of polymer-based sleeves is safe installation without taking the pipeline out of service. This paper presents a new calculation procedure proposed in the form of an algorithm for the sizing of composite repairs of corroded pipelines when the sleeve is applied at zero internal pressure. The main objective of the presented methodology is determination of the effective thickness of the composite repair without its overestimation or underestimation. The authors used a non-linear finite element method with constitutive models allowing analysis of the steel, putty, and composite structures. The validation of the results of numerical computations compared to the experimental ones showed an appropriate agreement. The numerical calculations were applied to compare the analytical results in relation to those obtained by the standards ASME PCC-2 or ISO/TS 24817. The comparison showed that the proposed solution confirmed its effectiveness in reducing the thickness of the sleeve significantly, thus, showing that the current industrial standards provide a considerably excessive composite wrap around the steel pipe corroded area, which leads to an unnecessary increase in the repair costs.

## 1. Introduction

Pipelines are the safest and most economical way to transport various hydrocarbon products over long distances. However, as they are made of steel, they tend to degrade in the corrosive environment. Furthermore, despite the availability of corrosion protection technologies, underground structures are still susceptible to degradation with time of operation [1,2,3]. During inspections, scientists and engineers frequently deal with the problem of tube wall metal loss, in particular, with its impact on the ability of the repaired thin-walled cylinder to withstand the design pressure [4]. For the unacceptable defects, in many service situations, the affected pipeline sections are removed and replaced with new ones, or in some cases, full-encirclement steel sleeves are welded around the corroded areas [5]. In both previously mentioned cases, such repairs entail significant costs due to the mandatory shutdown of the pipeline, as well as the materials and installation expenditures. Before proposing a composite overwrap as a new competitor to steel sleeves for repairs, their use had not been recognized by high pressure network operators despite its extensive application in many sectors, such as marine, automotive, and aerospace industries.

Since 2006, after the publication of international standards ASME PCC-2 [6] and ISO/TS 24817 [7], the polymer-based laminate has been recognized as a new competitor to steel sleeves for reinforcement of the tubes. As a result, the advantages of composite sleeves are widely accepted in pipeline renovation and have become a cost-effective approach due to their effectiveness in improvement of long-term solutions and installation safety, as well as in reduction of a risk of an unplanned shutdown [8]. Considering the above reasons, polymer-based materials for repairs of underground pressurized structures have become the subject of many publications. Regarding the development and qualification of selection of composite materials for pipeline renovations, such studies as [9,10,11,12,13,14,15,16] were analyzed. The research focuses on improving the existing methodologies or developing new ones, such as, but not limited to [17,18,19,20,21,22,23,24].

In regard to the design standards, the results of numerical and analytical research have shown that ISO/TS 24817 and ASME PCC-2 lead to greater thickness of sleeves for small and medium feature depths. In view of the foregoing, this paper presents a new analytical approach for providing the minimal thickness when the overwrap is applied at zero internal pressure. According to the authors’ best knowledge, it is the first time the effect of external pressure has been taken into account. The external pressure can be considered to be an important factor when repairing either trans-sea or buried onshore pipelines. In order to include the effect of the putty material on the thickness to be calculated, the concept of compound cylinders without shrinkage fit is used. The putty material is classified as an elastic isotropic material and the composite as elastic anisotropic material. The strain-hardening behavior is reached with the elasto-plastic stress–strain relationship of the steel pipe. To validate the effectiveness of the proposed design solution, a finite element analysis (FEA) approach was adopted in the present study.

## 2. Applied Methodologies

### 2.1. Standards ASME PCC-2 and ISO/TS 24817

With regard to ASME PCC-2 and ISO/TS 24817 standards [6,7], thickness $t_{c}$ of the reinforcement system can be calculated using the following general equation provided by:

(1) $$\varepsilon_{c}=\frac{P_{d}D_{ext}}{2E_{c}t_{c}}-S\frac{t_{s}}{E_{c}t_{c}}-\frac{P_{live}D_{ext}}{2\left(E_{c}t_{c}+E_{s}t_{s}\right)}$$

where$\;P_{live}$ is the internal pressure at the time of the composite repair application, and $P_{d}$ is the internal design pressure of the pipeline,$\;E_{c}$ and $E_{s}$ are the elasticity moduli in the circumferential direction of the laminate and steel, respectively,$\;t_{s}$ is the remaining thickness of the pipe wall in the corroded zone, and $t_{c}$ is the minimum required thickness of the sleeve,$\;D_{ext}$ is the outside diameter of the steel tube, and $\epsilon_{c}$ is the composite allowable strain.

It should be noted that the difference between the two methods is included in the definition of stress *S*: where *S* is defined as the specified minimum yield stress of steel, according to ASME PCC-2 code and as the admissible stress of the pipe wall according to ISO/TS 24817. When the composite repair system is applied at zero internal pressure$\;\left(P_{live}=0\right)$, Equation (1) could be reduced as follows:

(2) $$\varepsilon_{c}=\frac{P_{d}D_{ext}}{2E_{c}t_{c}}-S\frac{t_{s}}{E_{c}t_{c}}$$

However, ASME PCC-2 and ISO/TS 24817 standards neglect the putty material contribution and the strain-hardening effect.

### 2.2. Analytical Approach

**Figure 1:**
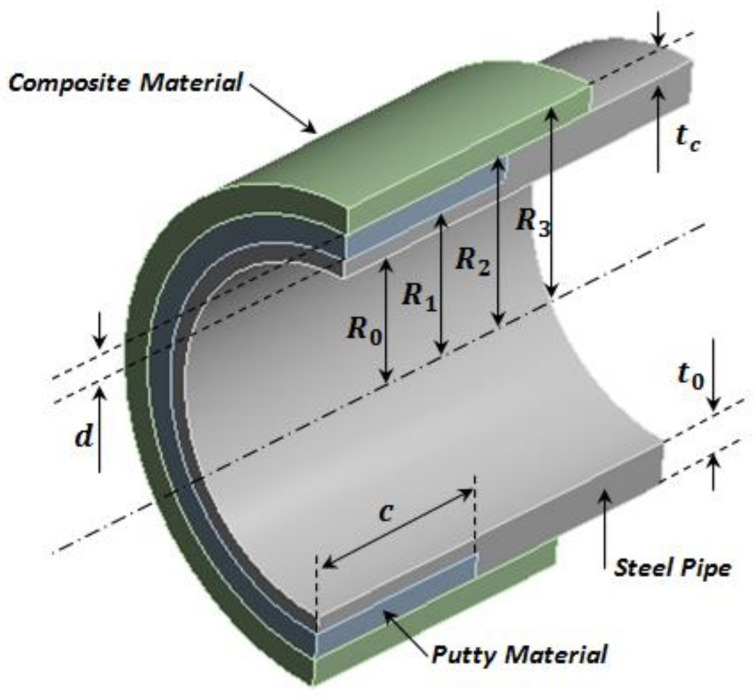
A typical composite overwrap repair system.

Hypothetically, if the thickness of the steel tube, shown in Figure 1, is $t_{0},$ and its internal radius is $R_{0}$, and on its outer side, there is a rectangular corrosion defect of length c, sufficient width w, and maximum depth d, in order to repair this flaw using a composite overwrap system, the cavity of the metal loss needs to be filled with a high-compressive-strength epoxy putty. Then, the polymer-based laminate is applied circumferentially until the desired thickness $t_{c}$ is obtained. The compound cylinder is subjected to internal pressure $P_{i}$ and to external pressure $P_{ext}$.

Therefore, the purpose of the sizing is to calculate the minimum thickness $t_{c}$ of the polymer-based sleeve to restore the load-bearing capacity of the damaged tube wall in order to withstand the internal design pressure $P_{d}$. When the pressure $P_{i}$ is applied, contact pressures are produced between three cylinders, shown in Figure 1. The contact pressures are as follows:$\;P_{c,1}\;$at $r=R_{1}$ (between the outer side of the steel and the inner side of the filling material) and the contact pressure $P_{c,2}\;$at $r=R_{2}$ (between the outer side of the putty material and the inner side of the composite material). At$\;r=R_{1}$, the steel pipe side and the putty material side are subject to stress in the circumferential, radial, and axial directions. The stress components can be found on each side of the cylinder using Lame’s relationships:

(3) $$\sigma_{\theta ,1}^{s}=\frac{R_{0}^{2}P_{i}-R_{1}^{2}P_{c,1}}{R_{1}^{2}-R_{0}^{2}}+\frac{\left(P_{i}-P_{c,1}\right)R_{0}^{2}}{\left(R_{1}^{2}-R_{0}^{2}\right)}$$

(4) $$\sigma_{r,1}^{s}=\frac{R_{0}^{2}P_{i}-R_{1}^{2}P_{c,1}}{R_{1}^{2}-R_{0}^{2}}-\frac{\left(P_{i}-P_{c,1}\right)R_{0}^{2}}{\left(R_{1}^{2}-R_{0}^{2}\right)}$$

(5) $$\sigma_{a,1}^{s}=\frac{R_{0}^{2}P_{i}-R_{1}^{2}P_{c,1}}{R_{1}^{2}-R_{0}^{2}}$$

and

(6) $$\sigma_{\theta ,1}^{p}=\frac{R_{1}^{2}P_{c,1}-R_{2}^{2}P_{c,2}}{R_{2}^{2}-R_{1}^{2}}+\frac{\left(P_{c,1}-P_{c,2}\right)R_{2}^{2}}{\left(R_{2}^{2}-R_{1}^{2}\right)}$$

(7) $$\sigma_{r,1}^{p}=\frac{R_{1}^{2}P_{c,1}-R_{2}^{2}P_{c,2}}{R_{2}^{2}-R_{1}^{2}}-\frac{\left(P_{c,1}-P_{c,2}\right)R_{2}^{2}}{\left(R_{2}^{2}-R_{1}^{2}\right)}$$

(8) $$\sigma_{a,1}^{p}=\frac{R_{1}^{2}P_{c,1}-R_{2}^{2}P_{c,2}}{R_{2}^{2}-R_{1}^{2}}$$

Moreover, at radius $r=R_{2}$, the stress components can be found on the outer surface of the filling material and on the inner surface of the composite material:

(9) $$\sigma_{\theta ,2}^{p}=\frac{R_{1}^{2}P_{c,1}-R_{2}^{2}P_{c,2}}{R_{2}^{2}-R_{1}^{2}}+\frac{\left(P_{c,1}-P_{c,2}\right)R_{1}^{2}}{\left(R_{2}^{2}-R_{1}^{2}\right)}$$

(10) $$\sigma_{r,2}^{p}=\frac{R_{1}^{2}P_{c,1}-R_{2}^{2}P_{c,2}}{R_{2}^{2}-R_{1}^{2}}-\frac{\left(P_{c,1}-P_{c,2}\right)R_{1}^{2}}{\left(R_{2}^{2}-R_{1}^{2}\right)}$$

(11) $$\sigma_{a,2}^{p}=\frac{R_{1}^{2}P_{c,1}-R_{2}^{2}P_{c,2}}{R_{2}^{2}-R_{1}^{2}}$$

and

(12) $$\sigma_{\theta ,2}^{c}=\frac{R_{2}^{2}P_{c,2}-R_{3}^{2}P_{ext}}{R_{3}^{2}-R_{2}^{2}}+\frac{\left(P_{c,2}-P_{ext}\right)R_{3}^{2}}{\left(R_{3}^{2}-R_{2}^{2}\right)}$$

(13) $$\sigma_{r,2}^{c}=\frac{R_{2}^{2}P_{c,2}-R_{3}^{2}P_{ext}}{R_{3}^{2}-R_{2}^{2}}-\frac{\left(P_{c,2}-P_{ext}\right)R_{3}^{2}}{\left(R_{3}^{2}-R_{2}^{2}\right)}$$

(14) $$\sigma_{a,2}^{c}=\frac{R_{2}^{2}P_{c,2}-R_{3}^{2}P_{ext}}{R_{3}^{2}-R_{2}^{2}}$$

From Equations (4) and (7), the radial stress of both materials, namely, the steel of the pipe and the putty filler, at the contact interface $r=R_{1}\;$are defined as follows:

(15) $$\sigma_{r,1}^{s}=-P_{c,1}$$

(16) $$\sigma_{r,1}^{p}=-P_{c,1}$$

From Equations (10) and (13), the radial stress of the filler material and the composite sleeve material at the contact interface $r=R_{2}\;$are defined as follows:

(17) $$\sigma_{r,2}^{p}=-P_{c,2}$$

(18) $$\sigma_{r,2}^{c}=-P_{c,2}$$

In order to simplify the stress-pressure relationships, Equation (15) is substituted into Equation (4) and rearranged as follows:

(19) $$\frac{R_{0}^{2}P_{i}-R_{1}^{2}P_{c,1}}{R_{1}^{2}-R_{0}^{2}}=-P_{c,1}\left(1+\alpha_{0}\right)+\alpha_{0}P_{i}$$

where:

(20) $$\alpha_{0}=\frac{R_{0}^{2}}{\left(R_{1}^{2}-R_{0}^{2}\right)}$$

Similarly, Equation (16) is substituted into Equation (7):

(21) $$\frac{R_{1}^{2}P_{c,1}-R_{2}^{2}P_{c,2}}{R_{2}^{2}-R_{1}^{2}}=-P_{c,1}\left(1-\alpha_{1}\right)-\alpha_{1}P_{c,2}$$

where:

(22) $$\alpha_{1}=\frac{R_{2}^{2}}{\left(R_{2}^{2}-R_{1}^{2}\right)}$$

Additionally, Equation (17) is substituted into Equation (10):

(23) $$\frac{R_{1}^{2}P_{c,1}-R_{2}^{2}P_{c,2}}{R_{2}^{2}-R_{1}^{2}}=-P_{c,2}\left(1+\alpha_{2}\right)+\alpha_{2}P_{c,1}$$

where:

(24) $$\alpha_{2}=\frac{R_{1}^{2}}{\left(R_{2}^{2}-R_{1}^{2}\right)}$$

Finally, Equation (18) is substituted into Equation (13):

(25) $$\frac{R_{2}^{2}P_{c,2}-R_{3}^{2}P_{ext}}{R_{3}^{2}-R_{2}^{2}}=-P_{c,2}\left(1-\alpha_{3}\right)-\alpha_{3}P_{ext}$$

where:

(26) $$\alpha_{3}=\frac{R_{3}^{2}}{\left(R_{3}^{2}-R_{2}^{2}\right)}$$

Substituting Equations (19), (21), (23) and (25) into Equations (3)–(14), respectively, the following simplified expressions are obtained:

for the outer side of pipe steel:

(27) $$\sigma_{\theta ,1}^{s}=-\left(1+2\alpha_{0}\right)P_{c,1}+2\alpha_{0}P_{i}$$

(28) $$\sigma_{r,1}^{s}=-P_{c,1}$$

(29) $$\sigma_{a,1}^{s}=-\left(1+\alpha_{0}\right)P_{c,1}+\alpha_{0}P_{i}$$

for the inner side of the filler material:

(30) $$\sigma_{\theta ,1}^{p}=-\left(1-2\alpha_{1}\right)P_{c,1}-2\alpha_{1}P_{c,2}$$

(31) $$\sigma_{r,1}^{p}=-P_{c,1}$$

(32) $$\sigma_{a,1}^{p}=-\left(1-\alpha_{1}\right)P_{c,1}-\alpha_{1}P_{c,2}$$

for the outer side of the filler material:

(33) $$\sigma_{\theta ,2}^{p}=-\left(1+2\alpha_{2}\right)P_{c,2}+2\alpha_{2}P_{c,1}$$

(34) $$\sigma_{r,2}^{p}=-P_{c,2}$$

(35) $$\sigma_{a,2}^{p}=-\left(1+\alpha_{2}\right)P_{c,2}+\alpha_{2}P_{c,1}$$

for the inner side of the composite material:

(36) $$\sigma_{\theta ,2}^{c}=-\left(1-2\alpha_{3}\right)P_{c,2}-2\alpha_{3}P_{ext}$$

(37) $$\sigma_{r,2}^{c}=-P_{c,2}$$

(38) $$\sigma_{a,2}^{c}=-\left(1-\alpha_{3}\right)P_{c,2}-\alpha_{3}P_{ext}$$

Assuming that the hoop stress at the outer side of the pipe reaches a given yielding stress $\sigma_{\theta ,1}^{s}\;$for a given internal pressure $P_{i}$, the contact pressure $P_{c,1}\;$can be solved using Equation (27).

(39) $$P_{c,1}=-\frac{\sigma_{\theta ,1}^{s}-2\alpha_{0}P_{i}}{\left(1+2\alpha_{0}\right)}$$

In order to find the contact pressure $P_{c,2}$ at the interface $r=R_{2}$, it is assumed that the radial displacement is the same for both the filler material and the composite material $u_{r,2}^{p}=u_{r,2}^{c}$. If it is known that the hoop strain can be related to radial displacement at any radius r through as$\;\epsilon_{\theta }=u_{r}/r$, the following relationship can be written:

(40) $$\epsilon_{\theta ,2}^{p}=\epsilon_{\theta ,2}^{c}$$

As described previously, both the filler material and the composite material are supposed to be elastic. Thus, the hoop strains $\epsilon_{\theta ,2}^{p}\;$and $\epsilon_{\theta ,2}^{c}$ can be given as follows:

(41) $$\epsilon_{\theta ,2}^{p}=\frac{1}{E_{p}}\left[\sigma_{\theta ,2}^{p}-v_{s}\left(\sigma_{a,2}^{p}+\sigma_{r,2}^{p}\right)\right]$$

(42) $$\epsilon_{\theta ,2}^{c}=\frac{1}{E_{\theta }^{c}}\sigma_{\theta ,2}^{c}-\left(\frac{v_{r\theta }^{c}}{E_{r}^{c}}\sigma_{r,2}^{c}+\frac{v_{a\theta }^{c}}{E_{a}^{c}}\sigma_{a,2}^{c}\right)$$

Substituting Equations (33)–(35) into Equation (41) gives contact pressure $P_{c,2}$ in the following form:

(43) $$P_{c,2}=\frac{P_{c,1}\left(v_{p}-2\right)\alpha_{2}+E_{p}\epsilon_{\theta ,2}^{p}}{\alpha_{2}\left(v_{p}-2\right)+\left(2v_{p}-1\right)}$$

It should be mentioned that if the hoop strain $\epsilon_{\theta ,2}^{c}$ equals the allowable strain$\;\epsilon_{c}$ of the composite material, Equation (40) requires that$\;\epsilon_{\theta ,2}^{p}=\epsilon_{c}$. Thus, the contact pressure $P_{c,2}$ can be solved using Equation (43).

Regarding the thickness of the repair $t_{c}$, radius $R_{3}$ needs to be determined first. Hence, substituting Equations (36)–(38) into Equation (42) and rearranging the formula gives:

(44) $$\epsilon_{\theta ,2}^{c}=-P_{c,2}\left[\frac{1}{E_{\theta }^{c}}-\left(\frac{v_{a\theta }^{c}}{E_{a}^{c}}+\frac{v_{r\theta }^{c}}{E_{r}^{c}}\right)\right]+\alpha_{3}\left(P_{c,2}-P_{ext}\right)\left[\frac{2}{E_{\theta }^{c}}-\frac{v_{a\theta }^{c}}{E_{a}^{c}}\right]$$

Solving Equation (44) for geometric ratio $\alpha_{3}\;$gives:

(45) $$\alpha_{3}=\frac{\epsilon_{\theta ,2}^{c}+P_{c,2}\left[\frac{1}{E_{\theta }^{c}}-\left(\frac{v_{a\theta }^{c}}{E_{a}^{c}}+\frac{v_{r\theta }^{c}}{E_{r}^{c}}\right)\right]}{\left(P_{c,2}-P_{ext}\right)\left[\frac{2}{E_{\theta }^{c}}-\frac{v_{a\theta }^{c}}{E_{a}^{c}}\right]}$$

External radius of the composite sleeve $R_{3}$ as a function of the geometric ratio $\alpha_{3}$ and the external radius of the pipe $R_{2}$ is obtained using Equation (26):

(46) $$R_{3}=R_{2}\frac{\sqrt{\alpha_{3}\left(\alpha_{3}-1\right)}}{\left(\alpha_{3}-1\right)}$$

Assuming that $R_{3}=R_{2}+t_{c}$, Equation (46) can be rearranged to provide thickness $t_{c}$ of the composite polymer-based sleeve as:

(47) $$t_{c}=R_{2}\left[\frac{\sqrt{\alpha_{3}\left(\alpha_{3}-1\right)}}{\left(\alpha_{3}-1\right)}-1\right]$$

For the case in which terms $\left(v_{a\theta }^{c}/E_{a}^{c}\right)$ and $\left(v_{r\theta }^{c}/E_{r}^{c}\right)\;$can be neglected, compared to significance of the term $\left(1/E_{\theta }^{c}\right)$, Equation (45) can be simplified to:

(48) $$\alpha_{3}=\frac{E_{\theta }^{c}\epsilon_{\theta ,2}^{c}+P_{c,2}}{2\;\left(P_{c,2}-P_{ext}\right)}$$

From Equation (48), it can be seen that the geometric ratio$\;\alpha_{3}$ becomes mostly dependent on mechanical properties of the laminate material in the circumferential direction.

#### Procedure to Find the Thickness *t_c_* Based on the Developed Approach

**Figure 2:**
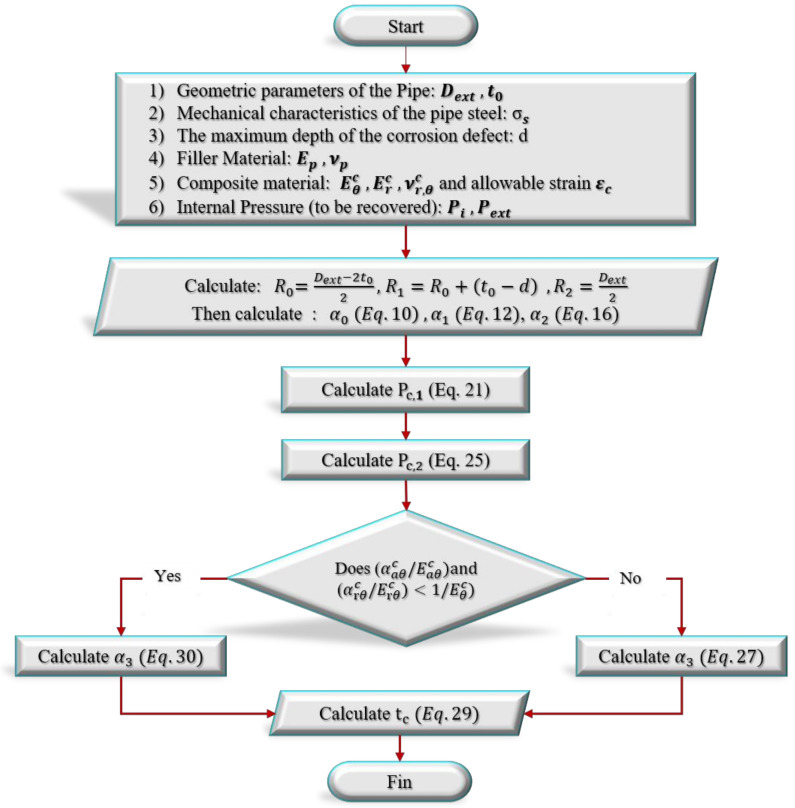
A flowchart for determining the thickness of a composite overwrap repair system.

In this section, a novel algorithm for designing the repair thickness $t_{c}$ of a corroded pipe using the composite material is presented. This solution is based on the formulas developed in the previous section. The steps of the proposed new approach are described in the flowchart in Figure 2.

If the pressure is substituted as $P_{i}=P_{d}$, the stress $\sigma_{\theta ,1}^{s}=K{\left(\epsilon_{\theta ,1}^{s}\right)}^{n}$ (n is the strain-hardening exponent) and the strain $\epsilon_{\theta ,2}^{c}=\epsilon_{c}$ are reached. The applied solution can be used to design a composite sleeve for recovering the initial design pressure $P_{d}$ of the pipeline. This pressure is also referred to as the maximum allowable working pressure (MAWP). $P_{d}$ is considered to be the relief pressure of the pipeline safety valves and is generally higher than the maximum operating pressure (MOP). According to Code B31.8 [25], $P_{d}$ is given as:

(49) $$P_{d}=FET\;\frac{2t_{0}}{D_{ext}}\;SMYS$$

where *F* is a design safety factor, *E* is a longitudinal welding joint factor and *T* is a temperature derating factor. In this paper, a value of 0.72 has been adopted for *FET*.

If the pressure is substituted as the burst pressure $P_{i}=P_{b}$, the plastic stress $\sigma_{\theta ,1}^{s}=K{\left(\epsilon_{\theta ,1}^{s}\right)}^{n}$ and the strain $\epsilon_{\theta ,2}^{c}=\epsilon_{c}$ are reached. The proposed approach provides the composite sleeve thickness for reconstructing the initial load-carrying capacity of the tube as burst pressure$\;P_{0\;}$ of an intact pipe. A decision to repair a corroded cylinder generally depends on its remaining load-carrying capacity, which should sufficiently withstand the design pressure. The remaining load-carrying capacity is carried out using ASME B31.G code [26]. Therefore, the burst pressure of a tube containing a metal loss is given as:

(50) $$P_{f}=\frac{2t_{0}}{D_{ext}}\left(SMYS+69\;\mathrm{MPa}\right)\frac{1-0.85\;\frac{d}{t_{0}}}{1-0.85\;\frac{d}{t_{0}}\frac{1}{M}}$$

where$\;z=c^{2}/(D_{ext}t_{0})$ is the normalized metal loss length, and M is the Folias factor:

(51) $$M=\left\{\begin{array}{cc} \sqrt{1+0.6275z-0.003375z^{2}}, & \;\;z\leq 0\\ 0.032z+3.3, & \;\;z>0 \end{array}\right.$$

A corrosion defect is considered to be acceptable when the calculated burst pressure is equal to or greater than the maximum value of the pressure set for the pipe ($MAOP=0.9\;P_{d}$) multiplied by a safety factor, usually not less than 1.25:

(52) $$P_{f}/MAOP\geq 1.25$$

### 2.3. Finite Element Analysis

**Figure 3:**
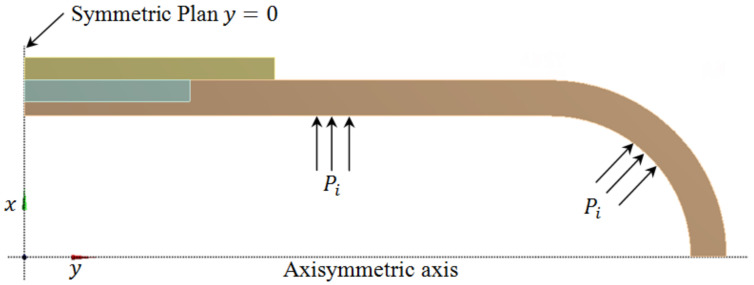
Repaired pipe with pressure load and boundary conditions.

While the steel tube and composite overwrap system are presumptive axisymmetric geometrical structures, the numerical model can be directly simplified into a 2D model. The key to this assumption is that the effect of the corrosion width is marginal and does not compromise the model accuracy [27,28]. As it is shown in Figure 3, the pipe and the repair sleeve are assumed to be axially symmetric around the central axis *y*. For this reason, an axisymmetric analysis was performed using two-dimensional 8-node quadrilateral elements (SOLID Plane 183) with the axisymmetric option activated. In addition, the pressure vessel is symmetric in respect to plane *y* = 0 running through the center of the cylindrical shell. Therefore, only a section of a quarter of the structure needs to be modeled.

The internal pressure $P_{i}$ is applied gradually, and when it reaches a value of $P_{i}\geq 0.9\;P_{recovered}$, the internal pressure increment ∆*P_i_* is insignificant and the numerical pressurization becomes slow. The calculation process stops when one of the two failure criteria is satisfied. These two criteria are the rupture criterion of the composite and the burst criterion of the pipe steel. The pressure obtained at the end of the calculation process is considered the maximum burst pressure.

**Figure 4:**
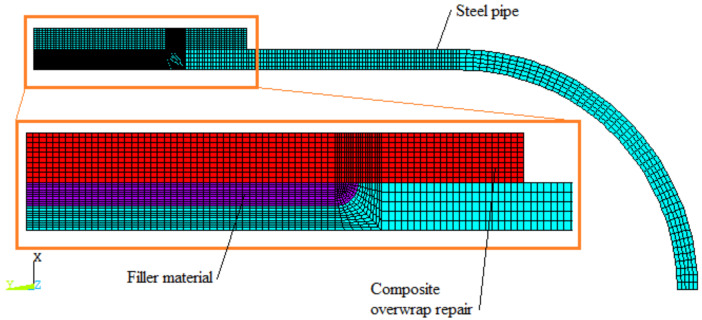
A finite elements mesh model with magnification of the repaired zone.

Figure 4 illustrates a mapped mesh of a steel tube, metal loss with the putty filler, and the composite overwrap. The irregular geometry of the model was divided into fully bonded sub-areas in order to control element sizing effectively. Then, mapped mesh with regular PLAN 183 quad elements was applied. A total of 4040 quadrilateral elements and 12,771 nodes were used.

## 3. Limit Load and Experimental Validation

To validate the numerical model as a viable method for the analysis of the pipeline repair system, experimental results found in the literature [18,23] were used due to the availability of all necessary data. Thus, one axisymmetric defect with 50% pipe wall thickness loss [18] and then one rectangular tube wall loss with 60% thinning [23] were machined into pipes. The first specimen was repaired using carbon–epoxy composites, and the second was repaired applying a fiber-glass epoxy material. Table 1 and Table 2 provide, respectively, the necessary engineering data for the used steel tube and the mechanical characteristics of the applied composites.

ANSYS APDL software was used to numerically simulate the burst tests of two defects of 50% and 60% tube wall thickness thinning. The engineering data presented in Table 1 and Table 2 were applied to calculations. Table 3 presents the predicted FEA and analytical burst pressures (ANA) of two flaws compared to the experimental results (EXP).

**Figure 5:**
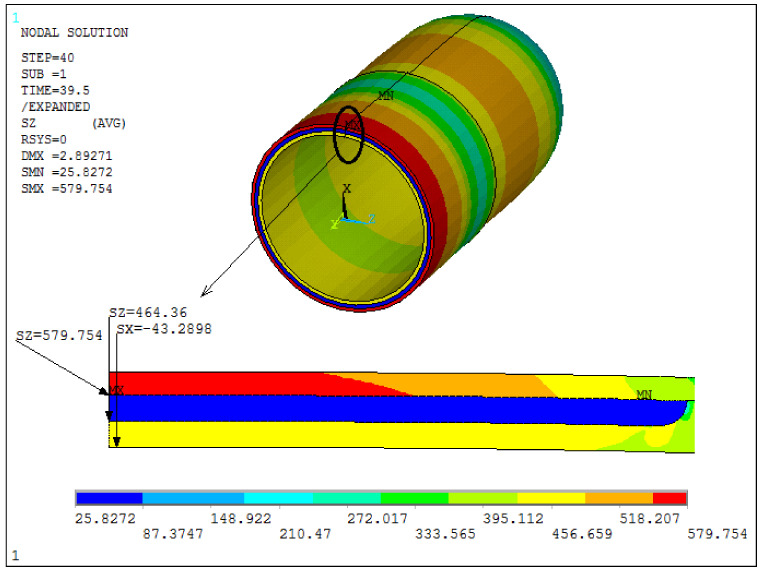
FEA predicted radial stress, steel and composite hoop stresses at the center of the 50% pipe wall loss axisymmetric defects, $P_{burst}=43.29\;\mathrm{MPa}$ (full expansion).

**Figure 6:**
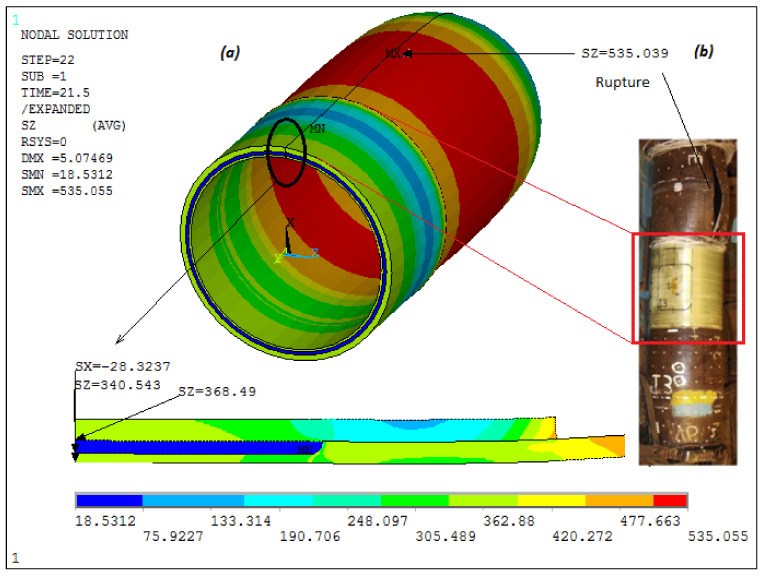
(**a**) FEA predicted radial stress, steel and composite hoop stresses at the center of the 60% pipe wall loss axisymmetric defects, $P_{burst}=28.32\;\mathrm{MPa}$, (**b**) comparison of the FEA predicted rupture zone with the experimental one performed by [23].

In Figure 5 and Figure 6, finite element analysis results for each defect at the predicted failure pressures are presented. Figure 5 shows that the highest failure inducing stresses occurred in the center of the axisymmetric defect region of 50% pipe wall loss depth. The failure pressure was determined when the circumferential stress of the composite reached its maximum value at 576 MPa, as provided in [18]. At the burst pressure, the hoop stress at the steel pipe reached 460 MPa. For the other case of the tube wall volumetric flaw with dimensions of 133 × 102 and 60% pipe wall loss depth, as can be seen in Figure 6a, the burst failure criterion was met in the steel outside the repair area. This criterion states that the tube wall fails when the circumferential stress of the pipe steel exceeds 530 MPa [23]; however, the stress in the composite remains below the failure stress (678 MPa). Figure 6b shows the comparison between the numerical simulation result and the experimental one [23] with respect to the rupture area. It was also noted that the analytical solution predicted that a repair thickness of 6 mm could increase the burst pressure to 46 MPa. Since the maximum internal pressure is 28.5 MPa, the rupture will occur outside the repair zone. In both cases, the predicted burst pressures, 43.29 MPa and 28.32 MPa, were close to the experimentally obtained values of 43.80 MPa and 29.06 MPa. Therefore, a good agreement between the results was obtained.

Furthermore, as can be seen in Table 3, for an unrepaired defected pipe, the resulting burst pressures for axisymmetric 152.4 × 152.4 flaw were 29.99 MPa and 30.34 MPa when the metal loss depth was 50%. As was mentioned above, the effect of feature width is marginal when the defect width is sufficient [27,28]. This fact can justify the use of an axisymmetric numerical model to analyze the repair of pipelines with the application of composite overwrap systems.

**Figure 7:**
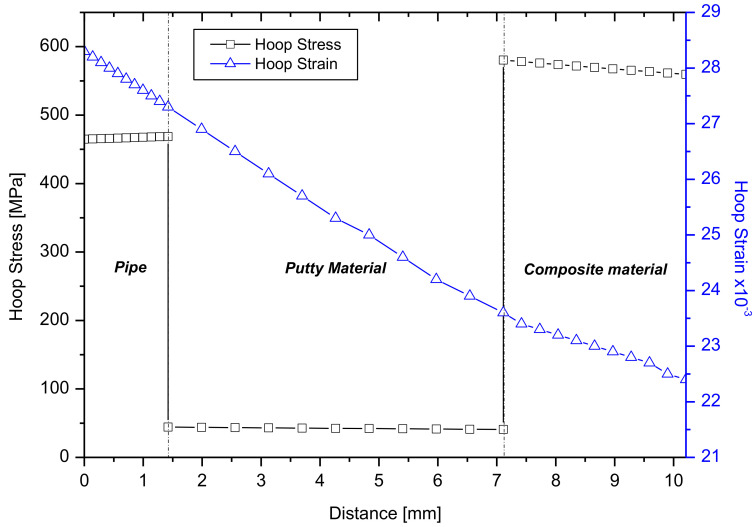
Evolution of circumferential stress and strain through the thickness for 50% wall loss axisymmetric defects.

Graphs of the circumferential stress and its corresponding strain at the center of the defect with dimensions of 152.4 × 152.4 in the cross section of materials were shown in Figure 7. There are two discontinuities in the stress–thickness plots as the material changes from steel to filler and from filler to CFRP. In both the examined cases, the failure criterion was reached first for the composite repair system. Although the failure pressures are not the same, the maximum stress and its corresponding strain (0.0235) were the same for both cases.

## 4. Composite Repair Sleeve Sizing at Design Pressure

In order to evaluate the validity of the suggested new solution for providing the minimum laminate sleeve thickness to effectively renovate a corroded pipeline at design pressure, a series of numerical representations of repairs were sized for corrosion features ranging in depth from 0.1$t_{0}$ to 0.9$t_{0}$. The wrap thickness $t_{c}$ was calculated using the developed approach, as well as the ASME PCC-2 code [6] and the ISO standard [7]. Consequently, using ANSYS software package, all repair systems resulting from each of the above methodologies were simulated. The purpose of the FEA is to estimate the hoop strain on the inner side of the composite sleeve and the outer side of the feature. Artificial metal losses were incorporated outside the pipe with the longitudinal length of features equal to 152.4 mm, axisymmetric widths, and uniform depths ranging from 10% to 90% of the wall thickness. Subsequently, the overwrap was applied circumferentially after filling defects with the putty filler. In the present analysis, the sleeve used for the reinforcement was assumed to be a fiber-glass reinforced epoxy composite. Table 4 provides the mechanical properties of the laminate and the filler material from work [29], respectively. It is assumed that the repair lifetime is 10 years, similarly as was adopted in [7]. However, the allowable circumferential laminate strain is 0.3%, as is assumed in Article 4 of ASME PCC-2.

## 5. Results and Discussion

**Figure 8:**
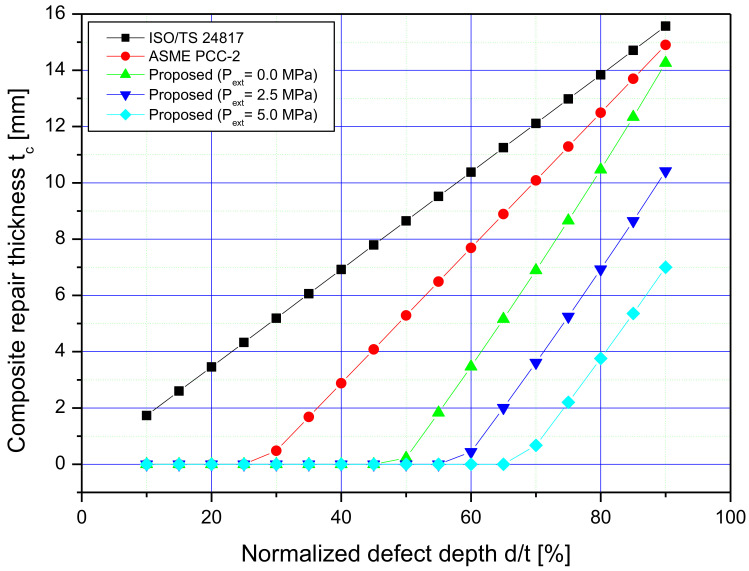
Composite thickness calculated analytically at the design pressure.

Table 5 and Figure 8 summarize and plot the analytical calculation results for the composite repair configurations considered in this paper. These values were obtained using the developed approach, ASME PCC-2, as well as ISO/TS 24817. All the reinforcements correspond to design conditions, including the design pressure of the tube (calculated as 14.68 MPa from Equation (49)), and the metal loss depths varying from 10 to 90% of the thickness of the intact pipe. From Figure 8, it can be seen that all three methods result in laminate thickness increasing with the metal loss radial size. For volumetric flaws with a depth d/t=10% to 90%, the ISO/TS-24817 standard resulted in excessive overwrap thickness compared to ASME PCC-2 and the proposed approach. The presented algorithm led to even smaller laminate thickness compared to the results of PCC-2 standard providing relatively small sleeve thickness. It should be highlighted that, in contrast to ISO/TS 24817 standard, both PCC-2 methodology and the proposed solution did not require application of composite repairs for the defect depths ranging from 25% to 45% of the intact tube. Taken into consideration the $P_{f}/MAOP$ ratio values, it is noted that the presented approach provides the sleeve thickness only when $P_{f}/MAOP\leq 1.25$. Therefore, it can be observed that the proposed solution provides the overwrap thickness for the metal losses with a 50% depth of the tube wall thickness, whereas, according to B31.G code, such corrosion anomalies are acceptable, however, without reinforcement. Thus, the results of sizing with a methodology developed in the current paper are fully consistent with that of the B31.G standard, recommending the reinforcement if the condition for the steel pipe wall loss, $P_{f}/MAOP<1.25,$ is not met. ISO/TS 24817 and ASME PCC-2 codes for sizing the composite sleeves lead to unnecessary repairs of steel tube wall volumetric flaws ranging from 10% to 50% and from 30% to 50%, respectively, for the two standards. Furthermore, the effect of external pressure $P_{ext}$, such as water hydrostatic pressure, on the thickness of the overwrap is shown in Figure 8. As can be observed, the thickness of the laminate decreases with the increasing external pressure around the tube. At $P_{ext}=2.5\;$MPa, a repair condition starts from a feature depth of 60% instead of 50%, as in the case of zero external pressure. In this case, the composite sleeve thickness is 0.44 mm instead of 3.47 mm. When increasing the external pressure to 5.0 MPa, it is possible to apply a polymer-based overwrap for defects with a depth starting from 70% instead of 50%, as in the case of zero external pressure, and a thickness of 0.67 mm instead of 6.89 mm. Therefore, for medium corrosion anomalies ranging from 50% to 65%, the proposed approach provides the sleeve thickness of 96% to 42% smaller than in the case of ASME PCC-2, and 32% to 4% smaller for deep metal losses (70% to 90%). With regard to ISO/TS 24817 for the external pressure of 5.0 MPa, the proposed method reduced the fiber-glass thickness from 97.5% to 54% for medium defects depths ranging from 50% to 65%. For the depth of pipe wall metal losses greater than 70%, the reduction of the sleeve thickness is by 43% to 8.4% compared to ISO/TS 24817 for the external pressure of 5.0 MPa. Unlike PCC-2 and ISO standards, the proposed solution does not provide any composite reinforcement for small depths of volumetric flaws ranging from 10% to 45%.

**Figure 9:**
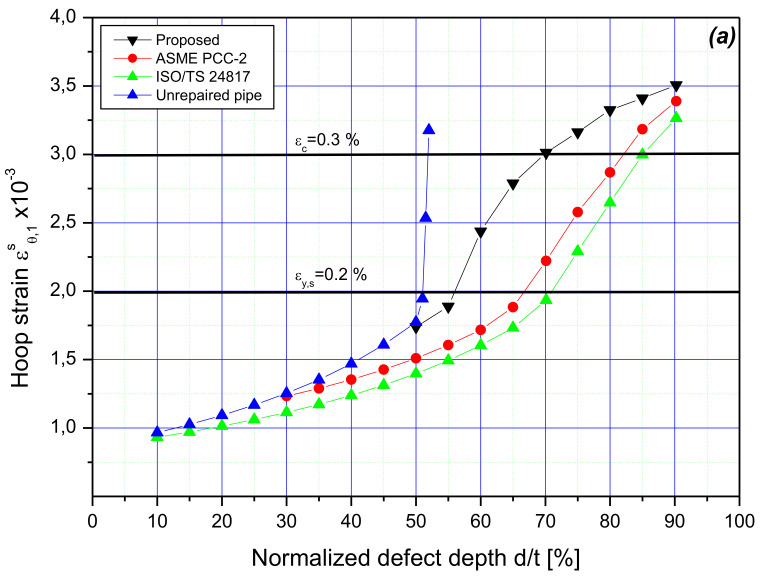

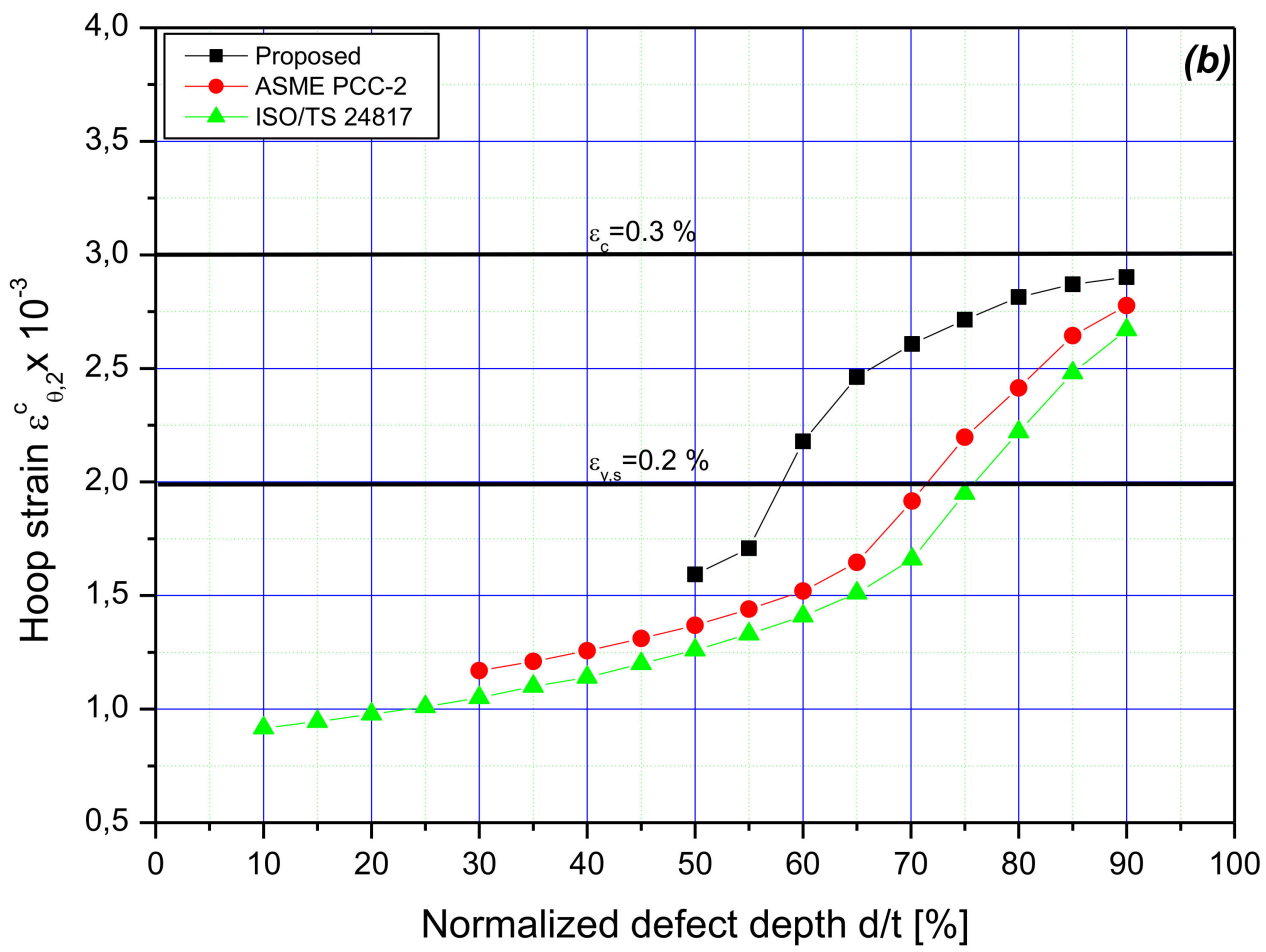
FEA predicted hoop strain at the design pressure: (**a**) outer surface of the pipe (middle of corrosion feature), (**b**) inner surface of the composite repair system.

The results presented in Figure 9 are hoop strain obtained from the finite element analysis at the design load of 14.68 MPa for depths of features ranging from 10% to 90%. Figure 9a shows the hoop strain$\;\epsilon_{\theta ,1}^{s}$ in the steel beneath the putty material, and Figure 9b shows the hoop strain $\epsilon_{\theta ,2}^{c}$ in the inner surface of the laminate. The most significant observation concerns the tube wall losses with a depth of 50%, whereas the developed methodology recommends repair when the defected steel pipe wall begins to plasticize. This confirms the above observation concerning the depth starting from which reinforcement of the flawed steel tube is necessary.

By comparing the obtained pipe hoop strains, it was clearly observed that the hoop strain, given by the developed method, in the corroded region was greater than those obtained by PCC-2 and ISO. The deformation given by the presented approach increased considerably with an increase in the metal loss depth and slightly exceeded the elastic limit of the steel. It should be noted that the developed calculation procedure tolerates a certain rate of strain-hardening for the steel material. For all the simulations, the stress is assumed to be 320 MPa. On the other hand, both PCC-2 and ISO standards increased with the defect depth in a similar way with a small deviation. This deviation depends on the allowable stress taken in the design, which is equal to the value of SMYS for PCC-2 and 0.72 SMYS for ISO. It can also be noted that, for these methods, the steel behavior is assumed to be elastic perfectly plastic; however, the steel has exceeded its allowable stress.

As shown in Figure 9b, when the volumetric flaw depth increased beyond 50%, plasticity was induced in the corroded zone of the steel pipe, and the mechanical load (internal pressure in the considered case) carried by the corroded area was transferred to the laminate through the filler material. As the feature depth increased, the strain of sleeve increased while remaining less than the allowable strain,$\;\epsilon_{c}=0.3\%\;,$ for polymer-based material. Although all methods presented approximately the same strain regarding the deepest defects, the hoop strain obtained with the developed algorithm was important for a medium depth of wall material losses. This was due to the efficiency of the proposed method to give smaller repair thickness while providing a laminate material strain lower than the admissible one.

## 6. Conclusions

In this paper, an analytical formulation and FEA studies were conducted in order to present a new cost-effective method for determining the composite sleeve thickness for the corroded pipe repairs. The analytical procedure proposed in the form of an algorithm is validated using experimental data taken from the literature. The validation of the methodology showed that this method can predict economical and efficient composite repair thicknesses at limit load conditions. The proposed algorithm is compared to ASME, PCC-2 and ISO/TS 2481 standards using the results of a series of finite element analyses. By analyzing the obtained results, the following conclusions can be drawn:

1. The composite overwrap sizing calculated according to ASME PCC-2 and ISO/TS 2481 standards is conservative with respect to the obtained sleeve thickness and results in unnecessary repairs of the steel pipe wall thinning, particularly for small depths of material losses. For a medium depth of metal losses, ranging from 50% to 65% of the tube wall, in the case of the new developed solution, the sleeve thickness was reduced as follows:
  - by 96% to 42% compared to the results of ASME PCC-2,
  - by 97.5% to 54% compared to the results of ISO/TS 2481.
2. For the deepest pipe wall metal losses, starting from 70% of wall thickness, the applied sizing algorithm reduces the wrap thickness as follows:
  - by 32% to 4% compared to the results of ASME PCC-2,
  - by 43% to 8.4% compared to the results of ISO/TS 2481.
3. The presented methodology takes into consideration an effect of the external pressure surrounding the tube, as in the case of offshore pipelines, in which the thickness of the fiber-glass sleeve is even less.
4. The proposed approach predicts the burst pressure of the defected pipes repaired with a composite overwrap system for practical applications. Due to this fact, the authors are going to conduct experiments to validate the burst pressure of steel tubes repaired with the use of the developed sleeve sizing procedure.

## Figures and Tables

**Table 1:** Pipe material properties.

Reference	[18]	[23]
Steel grade	A106 Gr B	GOST 8731-74
External diameter $D_{ext}$ (mm)	152.3	220
Pipe wall thickness t (mm)	7.11	6.0
Yield stress$\;\sigma_{y}$ (MPa)	300 (0.2% offset)	305 (0.2% offset)
SMYS (MPa)	241.3	241.3
Poisson’s ratio$\;v_{s}$	0.30	0.30
Hollomon true σ−ϵ model	$823.33\epsilon^{0.1813}$	$697\epsilon^{0.136}$

**Table 2:** Laminate and filler material mechanical properties.

Reference	[18]	[23]
Polymer-based laminate	Carbon fiber/epoxy	Glass fiber/epoxy
Modulus in the hoop direction $E_{\theta }$ (MPa)	49,000	48,470
Modulus in the axial direction $E_{a}$ (MPa)	23,400	6770
Modulus in the radial direction $E_{r}$(MPa)	5500	6770
Poisson’s ratio $v_{ra}$	0.45	0.4
Poisson’s ratio $v_{a\theta }$	0.07	0.099
Poisson’s ratio $v_{r\theta }$	0.45	0.099
Shear modulus $G_{ra}$ (MPa)	690	1670
Shear modulus $G_{a\theta }$ (MPa)	2960	3200
Shear modulus $G_{r\theta }$ (MPa)	690	3200
Failure stress in hoop direction (MPa)	576	678
Composite thickness $t_{c}$ (mm)	3.1	6
**Filler Material**		
Young’s Modulus $E_{p}$ (MPa)	1740	3300
Poisson’s ratio$\;v_{p}$	0.45	0.37

**Table 3:** Comparison of repaired tubes.

Defect Type Length (mm) × Width (mm)	Flaw Depth d/t (%)	Burst Pressure, MPa (Unrepaired)	EXP Burst Pressure, MPa (Repaired)	FEA Burst Pressure, MPa (Repaired)	ANA Burst Pressure, MPa (Repaired)	Reference
Intact steel pipe (Unflawed)	0%	45.85	N/A	N/A	N/A	[18]
Axisymmetric	50%	29.99	43.80	43.29	44.15	
152.4 × 152.4	50%	30.34	43.10	N/A	44.15	
Intact steel pipe (Unflawed)	0%	27.59	N/A	N/A	N/A	[23]
133 × 102	60%	13.8	29.06	28.32	46.4	

**Table 4:** Laminate and filler material mechanical properties.

Polymer-Based Laminate	
Modulus in the hoop direction $E_{\theta }$ (MPa)	23,800
Modulus in the axial direction $E_{a}$ (MPa)	24,500
Modulus in the radial direction $E_{r}$(MPa)	11,600
Poisson’s ratio $v_{ra}$	0.071
Poisson’s ratio $v_{a\theta }$	0.107
Poisson’s ratio $v_{r\theta }$	0.1
Shear modulus $G_{ra}$ (MPa)	2600
Shear modulus $G_{a\theta }$ (MPa)	4700
Shear modulus $G_{r\theta }$ (MPa)	3600
Laminate allowable strain $\epsilon_{c}$(mm/mm)	0.003
**Filler Material**	
Young’s Modulus $E_{p}$ (MPa)	1.740
Poisson’s ratio$\;v_{p}$	0.45

**Table 5:** Sizing of composite sleeve thickness as a function of relative metal loss depth.

*P_d_* [MPa] (Equation (49))	$\frac{d}{t}\;[\%]$	$\frac{P_{f}}{MAOP}$	$t_{c}\;[\mathrm{mm}]$				
			ASME PCC-2 (S = SMYS)	ISO/TS24817 (S = 0.72 SMYS)	$\mathrm{Developed}\;(P_{ext}=0\;\mathrm{MPa})$	$\mathrm{Developed}\;(P_{ext}=2.5\;\mathrm{MPa})$	$\mathrm{Developed}\;P_{ext}=5\;\mathrm{MPa}$
14.68 (Pressure $P_{i}$ to be recovered)	10	1.86	0	1.73	0	0	0
	15	1.79	0	2.60	0	0	0
	20	1.73	0	3.46	0	0	0
	25	1.66	0	4.33	0	0	0
	30	1.59	0.48	5.19	0	0	0
	35	1.52	1.68	6.06	0	0	0
	40	1.45	2.88	6.92	0	0	0
	45	1.37	4.08	7.79	0	0	0
	50	1.30	5.29	8.65	0.22	0	0
	55	1.22	6.49	9.52	1.83	0	0
	60	1.14	7.69	10.38	3.47	0.44	0
	65	1.05	8.89	11.25	5.16	2.01	0
	70	0.97	10.09	12.11	6.89	3.61	0.67
	75	0.88	11.29	12.98	8.66	5.25	2.2
	80	0.79	12.49	13.84	10.47	6.93	3.76
	85	0.69	13.70	14.71	12.34	8.65	5.36
	90	0.59	14.90	15.57	14.26	10.42	7.00

## Data Availability

All data generated or analyzed during this study are included in this published article.

## Nomenclature

$\;D_{ext}$	Outside diameter of the steel tube
$P_{recovered}$	Pressure to be recovered
$P_{d}$	Internal design pressure of the pipeline
$P_{live}$	Live pressure
$P_{i}$	Actual internal pressure
$P_{ext}$	External pressure
$\;P_{c,j}$	Contact pressure at the location j
$P_{f}$	Burst pressure of the steel pipe
$t_{c}$	Minimum required thickness of the composite sleeve
$\;t_{s}$	Remaining thickness of the pipe wall in the corroded zone
$t_{0}$	Thickness of the steel pipe
$\epsilon_{c}$	Composite allowable strain
S	Specified minimum yield stress of steel
n	Strain hardening exponent
K	Material strength coefficient
$R_{0}$, $R_{1},\;R_{2\;}\;\mathrm{and}\;R_{3}$	Internal radius of the steel tube, internal radius of corrosion defect, external radius of steel tube, and internal radius of composite repair
$\alpha_{0},\;\alpha_{1},\;\alpha_{2}\;\mathrm{and}\;\alpha_{3}$	Dimensionless geometric ratio
$\;\sigma_{y}$	Actual yield stress
$\sigma_{\theta ,i}^{j},\;\sigma_{a,i}^{j}\;\mathrm{and}\;\sigma_{r,i}^{j}$	Stress components, respectively, in hoop, axial, and radial directions. The indices *i* and j denote, respectively, the contact pressure location and the material type
$\epsilon_{\theta ,i}^{j},\;\epsilon_{a,i}^{j}\;\mathrm{and}\;\epsilon_{r,i}^{j}$	Strain components, respectively, in hoop, axial, and radial directions. The indices *i* and j denote, respectively, the contact pressure location and the material type
$E_{\theta }^{c},\;E_{a}^{c}\;\mathrm{and}\;E_{r}^{c}$	Elastic moduli of the composite, respectively, in the circumferential, axial, and radial directions
$v_{ra}^{c},v_{a\theta }^{c}\;\mathrm{and}\;v_{r\theta }^{c}$	Composite material Poisson’s ratio
$G_{ra}^{c},G_{a\theta }^{c}\;\mathrm{and}\;G_{r\theta }^{c}$	Shear moduli of the composite, respectively, in the circumferential, axial, and radial directions.
$v_{p}$	Putty Poisson’s ratio
c	Corrosion defect half length
w	Corrosion defect width
d	Corrosion defect depth
z	Normalized defect length
M	Folias factor
FET	Safety factors
MAWP	The maximum allowable working pressure
MOP	The maximum operating pressure
